# Associations between ethnicity, place of residence, hearing status of family and habilitation of children with hearing impairment

**DOI:** 10.1186/s13584-020-00394-1

**Published:** 2020-07-13

**Authors:** Ester Goldblat, Dori Rivkin, Viacheslav Konstantinov

**Affiliations:** 1 Administration of Disabilities, Ministry of Labor, Social Affairs & Social Services, P.O.B 1260, Jerusalem, Israel; 2 Family Group, Myers-JDC-Brookdale, Jerusalem, Israel

**Keywords:** Hearing loss, Cochlear implant, Habilitation, Mode of communication, Ethnicity, Geographic locations

## Abstract

**Background:**

Hearing parents tend to have a strong preference for their deaf and hard-of-hearing children to acquire adequate speech, as opposed to use of sign language. Research reports the contribution of many variables to speech acquisition by children with hearing loss (HL). Yet, little is known about the association between ethnicity, place of residence, and hearing status of family members and mode of communication of young people with HL. The purpose of the present study was to examine whether mode of communication of young people with HL is associated with ethnicity, place of residence, and hearing status of family members.

**Method:**

Participants were young adults with sensory-neural severe to profound HL, either congenital or acquired prior to age 3. Only participants without additional disabilities were included. The data on participants were extracted from records of the Ministry of Labor, Social Affairs and Social Services in Israel. The data for each participant in the study included mode of communication, gender, use of assistive device, ethnicity, geographic place of residence, and presence of first-degree relatives with HL. Regarding participants with a cochlear implant (CI), age at implantation was documented as well.

**Results:**

Chi-square tests revealed significant associations between mode of communication and all of the study variables. In addition, all the study variables made a significant contribution to mode of communication. Regarding ethnicity, most of the ultra-Orthodox participants used oral language, while the majority of Israeli-Arab participants used sign language. Regarding geographical place of residence, lower rates of oral language use were found in the northern and southern districts of Israel.

**Conclusions:**

The findings of the present study underline the need for better monitoring of Israeli-Arab children with HL and children residing in peripheral areas in Israel and for improving access to habilitation services.

## Background

Prelingual hearing loss is a hearing impairment that manifests before speech acquisition [1]. Hearing loss (HL), whether present at birth or occurring in childhood, often represents a significant barrier to spoken language development [2]. Thus, one of the main goals of habilitation programs for children with HL is the proper acquisition of oral language.

People with HL use oral, manual or combined communication. Oral communication refers to the use of spoken language. Manual communication refers to the use of sign language. Combined communication usually refers to the simultaneous use of oral and manual communication [3].

Children with profound HL are eligible for cochlear implants (CI) from the age of 12 months [4]. Most parents with normal hearing would like their child who is deaf to learn to communicate using spoken language and choose to provide a CI to facilitate this outcome [5]. Thus, one of the first decisions hearing parents must make after a diagnosis of HL in their young child concerns communication and language development [6].

Hearing parents tend to have a strong preference for their deaf and hard-of-hearing children to acquire adequate speech, to promote the inclusion of their children in the hearing world. Some of them appreciate sign language as the means of access to the Deaf community [6]. However, most hearing parents do not know sign language when their child is diagnosed with HL, and acquiring proficiency in this mode of communication is a long and arduous process for them [5].

Many variables are associated with better language development among children using CI. Among them are female gender, absence of additional disabilities, and age at implantation [7]. The mode of communication is significantly related to age at implantation, with age 3 serving as the cut-off point [8]. Demographic variables such as ethnicity and geographic residence have received little attention in studies on hearing impairment in general and CI specifically, although it has been documented that ethnic differences and place of residence seem to contribute to inequities in service use [9]. In other words, ethnicity may be a predictor for habilitation of children with hearing impairment.

Studies addressing the association between ethnicity and prelingual HL have reported that failure of parents to follow through with recommended care for their hearing-impaired newborns is predicted by race or ethnicity [10–12]. Pretto [13] reported that toddlers with HL from non-White families are diagnosed and habilitated later than children from White families. Yet little is known about the association between mode of communication of children with a CI and their racial-ethnic background [4].

### Minorities in Israel

One of the main ethnic groups in Israel is the Arab population. Israeli Arabs are considered a cultural group, and form the largest linguistic minority in Israel, with Arabic as the main language of its members [14]. Israeli Arabs also constitute the largest overall minority group in the country, comprising about 20% of the population [15]. Despite universal coverage and a national health insurance law in Israel in force since 1995, the wide array of gaps in health care service use has been extensively documented [16]. Research indicates that low health literacy is more common among Israeli Arabs (55%) than among Hebrew speakers (only 31%) [17]. In addition, fewer annual visits to specialists are reported for Israeli Arabs than for Jewish Israelis [18]. In general, Israeli Arabs have a problematic or inadequate level of health literacy, whereas the Jewish population in general has sufficient health literacy. This difference might be due to socioeconomic differences between the two groups rather than ethnic or cultural differences [19].

Marriages within extended families among the Arab population lead to a high prevalence of recessive traits, including HL [20, 21]. Parving & Hauch [22] reported a prevalence ranging from 1.35–1.91 children with HL for every 1000 live births. Only 15% percent of them were considered to have profound hearing impairment. In Israel, certain Bedouin tribes and Israeli Arabs as a whole exhibit a high prevalence of profound prelingual hereditary recessive deafness [23–25], extending up to 2.6–3.3% of profound prelingual HL [25]. Thus, Israeli Arab children with HL constitute a major group deserving habilitation services.

Another minority in Israel is the ultra-Orthodox Jewish population. Studies have related to this population, especially Hasidic Jews, as an ethnic group [26], as we do in the present study. Members of ultra-Orthodox communities refer to their religious leaders for guidance and advice on matters not necessarily related to religious practice [27], including health related issues [28]. Research demonstrates that this population makes lower use of certain health services, such as dental visits [29] or bone density examination [30]. Their health knowledge and awareness appears to be low compared with the general population in different fields [29–31].

Religiosity is assumed to play an important role in the lives of parents raising a child with HL and may contribute to their parenting behaviors and practices, as was revealed in a study conducted in Israel [32]. These specific findings shed light on habilitation of children with HL from ultra-Orthodox families, but this specific ethnic group deserves more attention in relation to ethnicity and hearing impairment.

Place of residence has not been the focus of research on HL habilitation. In general, socioeconomic inequities in health care use appear to be stronger in rural areas [9]). Most studies do not include place of residence as a variable associated with CI outcomes [4], though geographic place of residence has been found to have an impact on follow-up/non-follow-up in newborn hearing screening [11]. Thus, it is important to include geographical place of residence as a variable related to habilitation outcomes of children with HL. Finally, in relating to mode of communication it is important to consider the hearing status of the family. Children of deaf sign-language users are assumed to learn sign language through natural exposure to their parents’ native language [33]. Indeed, having at least one deaf parent is the dominant indicator for use of sign language by deaf and hard-of-hearing youth [34]. On the other hand, hearing parents find it difficult to learn sign language. They prefer hearing and speech as their child’s communication mode [35]. Thus, research on modes of communication among people with HL should relate to the hearing status of other family members as a related variable.

### The study

The purpose of the present study was to examine how demographic and family variables are related to modes of communication of young people with HL. Specifically, we explored whether mode of communication is related to ethnicity, place of residence, and hearing status of family members. Variables related to language development among children with a CI, such as gender and age at implantation, were considered as well.

The following hypotheses were tested: 1) The mode of communication will be related to ethnicity; 2) The mode of communication will be related to geographical place of residence; and 3) The mode of communication will be related to the hearing status of family.

## Method

### Participants and procedure

Participants were young adults with sensory-neural severe to profound HL, either congenital or acquired prior to age three. Only participants without additional disabilities were included.

The data on participants were extracted during 2019 from records of the Ministry of Labor, Social Affairs and Social Services (MOLSASS) in Israel after the researchers had received approval from the Ministry’s Research Division. The Ministry’s Disabilities Administration provides a variety of services for people with severe and profound HL, including material services that are provided regardless of the consumers’ social status. Eligibility for the services includes age of onset and severity of HL. Thus, the entire population of people with severe and profound HL who meet the criteria can receive material services. Due to the economic incentive, it is logical to assume that the majority of people in Israel with severe or worse HL are known to MOLSASS, as has been reported for parallel services [36]. The data for each participant in the study included mode of communication, gender, use of assistive device, ethnicity, geographic place of residence (geographic district), and presence of first-degree relatives with HL. Due to rapid advances in technology and the ramifications this has for habilitation of children with HL [37], we divided the entire sample into two age groups: 18–24 and 25–30. Regarding participants with a CI, age at implantation was documented as well.

### Statistical analysis

In order to examine associations between mode of communication and study variables (gender, age, ethnicity, assistive device, district, and presence of first-degree relatives with HL) chi-square tests were performed for the entire sample. In order to reveal relations between mode of communication and study variables among CI users chi-square tests were performed. Age at implantation was added as a study variable related to mode of communication among CI users, dividing participants with a CI into two groups: participants implanted before age 3 and participants who received their implants at age 3 and above.

In order to evaluate the contribution of study variables to mode of communication for the entire sample, and among the sub-group of participants using a CI, logistic multi-nominal regressions were performed. All of the study variables were entered into the regressions. Districts were grouped into two categories: the periphery consisting of the northern and southern districts, and the central area consisting of all other districts.

## Results

A total of 1210 young adults, aged 18 to 30 (*M* = 24.74, *Sd* = 3.54), with sensory-neural severe to profound HL, either congenital or acquired prior to age 3, participated in the present study. Six hundred seventy-nine of them (56.1%) were male and 531 (43.9%) were female. Regarding participants’ age, the entire sample was divided into two age groups: a younger group aged 18–24 and an older group aged 25–30. Five hundred fifty-three participants (45.7%) belonged to the younger group, and 657 participants (54.3%) belonged to the older group. Altogether 386 participants (31.9%) in the entire sample had a CI. The mean age of participants in this sub-group was lower than the mean age of the entire sample (*M* = 23.68, *Sd* = 3.50). Age at implantation was available only for 193 participants, ranging from 1 to 22 years of age (*M* = 6.87, *Sd* = 5.15). Participants who did not use CI either used hearing aids (592 participants, 48.9% of the entire sample) or did not use any hearing device (232 participants, 19.2% of the entire sample). Regarding mode of communication, 439 participants (36.3%) used oral language, 413 participants (34.1%) used manual communication, and 358 participants (29.6%) used combined communication. Regarding ethnicity, 460 participants (38.0%) were Israeli Arabs, 92 participants (7.6%) were Jewish Ultra-Orthodox, and 658 participants (54.4%) were from the general population. Regarding place of residence, we related to major cities and different districts in Israel. One hundred twenty-eight participants (10.6%) resided in Tel Aviv. One hundred forty-three participants (11.8%) resided in Haifa. One hundred ninety-two participants (15.9%) resided in Jerusalem. Two hundred forty participants (19.8%) resided in the central district. Two hundred forty-nine participants (20.6%) resided in the northern district. One hundred sixty-one participants (13.3%) resided in the southern district. Thirty-three participants (2.7%0 resided in Judea and Samaria. Sixty-four participants (5.3%) resided in the district of Ashdod and Ashkelon. Finally, regarding first-degree relatives with HL, data on this variable was available for only 1056 participants in the entire sample. Five hundred fifty-six participants (52.7% of participants with viable data) had first-degree relatives with HL, and 500 participants (47.3% of participants with viable data) did not have first-degree relatives with HL.

In order to examine associations between mode of communication and study variables (gender, age, ethnicity, assistive device, district, and presence of first-degree relatives with HL) chi-square tests were performed. Table 1 presents the results for the variables tested.

**Table 1:** Associations between mode of communication and study variables (in percentages)

Variable	Oral Communication (*n* = 439)	Manual Communication (*n* = 413)	Combined Communication (*n* = 358)	*Df*	*χ*^2^
Gender					
Male	34.9	37.1	28.0	2	6.183*
Female	38.0	30.3	31.6		
Age					
18–24	35.8	30.9	33.3	2	7.816*
25–30	36.7	36.8	26.5		
Ethnicity					
General	43.9	22.8	33.3	4	262.402**
Ultra-Orthodox	85.9	2.1	12.0		
Arab	15.4	56.7	27.8		
District					
Tel Aviv	44.5	21.9	33.6	14	182.794***
Center	43.3	22.1	34.6		
Haifa	32.2	29.4	38.5		
North	27.7	41.4	30.9		
Jerusalem	47.9	25.5	26.6		
South	10.6	73.3	16.1		
Judea/Samaria	69.7	12.1	18.2		
Ashdod/Ashkelon	48.4	25.0	26.6		
First-degree relatives with HL					
Yes	27.3	40.1	32.6	2	55.309***
No	46.2	21.4	32.4		
Assistive device					
CI	53.6	14.0	32.4	4	295.155***
Hearing aid	36.1	29.7	34.2		
No device	7.8	78.9	13.3		

*p* < .001*** *p* < .01** *p* < .05*

As shown in Table 1, there were significant differences in the mode of communication for all of the study variables. Oral communication was more common among female participants than among male participants, while sign language was more common among the male participants than among the female participants. Sign language was more common among the older participants than among the younger ones. Most ultra-Orthodox participants used oral language, while the majority of Arab participants used sign language. The majority of CI users used oral communication, while most participants with no hearing device used sign language. As to place of residence, we will only note the striking finding that most participants who lived in the southern district used sign language. Among participants who did not have first-degree relatives with HL, oral language was the common mode of communication, whereas among participants with first-degree relatives with HL, sign language was the common mode of communication.

Participants with a CI were classified as a sub-group of interest in the present study. In order to examine the associations between mode of communication and the study variables within this sub-group, chi-square tests were performed. Table 2 presents the results for the variables tested.

**Table 2:** Associations between mode of communication and study variables among CI users (in percentages)

Variable	Oral communication (*n* = 207)	Manual Communication (*n* = 54)	Combined Communication (*n* = 125)	*Df*	*χ*^2^
Gender					
Male	50.0	17.3	32.7	2	4.933*
Female	58.1	9.9	32.0		
Age					
18–24	50.0	13.5	36.5	2	4.512
25–30	59.0	14.7	26.3		
Ethnicity					
General	55.8	10.4	33.8	4	68.213***
Ultra-Orthodox	84.4	1.6	14.1		
Arab	32.2	34.1	42.7		
Age at implantation					
>3	70.7	4.9	24.4	2	4.232
≤3	55.9	15.8	28.3		
District					
Tel Aviv	61.7	10.6	27.7	14	42.444***
Center	58.7	8.7	32.6		
Haifa	45.7	5.7	48.6		
North	35.6	27.1	37.3		
Jerusalem	61.5	15.4	23.1		
South	20.0	32.0	48.0		
Judea/Samaria	75.0	0.0	25.0		
Ashdod/Ashkelon	64.7	5.9	29.4		
First-degree relatives with HL					
Yes	44.5	16.8	38.7	2	7.941**
No	59.3	12.1	28.5		

*p* < .001*** *p* < .01** *p* < .05*

As shown in Table 2, there were significant differences in the mode of communication of participants with a CI by gender, ethnicity, district, and presence of first-degree relatives with HL. A greater proportion of girls with a CI used oral language than did the boys, whereas more boys used sign language. The majority of ultra-Orthodox participants with a CI used oral language, while the highest proportion of sign language users was observed among Arab CI users. When comparing districts, the highest proportion of oral communication users was observed in Judea and Samaria, while the highest proportion of sign language speakers was observed in the southern part of the country. As to first-degree relatives with HL, a greater proportion of CI users without first-degree relatives with HL used oral language than CI users who had first-degree relatives with HL. A greater proportion of CI users with first-degree relatives with HL used sign language than CI users who did not have first-grade relatives with HL. No significant differences in mode of communication were found by age and age at implantation.

Since age at implantation was known for only 193 of the 386 participants with a CI, we used categorical variables by grouping categories of mode of communication. Firstly, we grouped manual communication and combined communication together in a single category and compared it with oral communication. For participants implanted before age three, 29.3% used manual communication or combined communication, and 70.7% used oral communication. For participants implanted at age three or older, 44.1% used manual communication or combined communication, and 55.9% used oral communication (*N* = 291, *x*^2^ = 2.930, *p* < .05). Thus, when we grouped the manual and combined communication modes into one category there were significant differences in mode of communication by age at implantation.

Next, we grouped oral and combined communication into one category and compared it with manual communication. For participants implanted before age three, 95.1% used oral communication or combined communication, and 4.9% used manual communication. For participants implanted at age three or older, 84.2% used oral communication or combined communication, and 15.8% used manual communication (*N* = 191, *x*^2^ = 3.298, *p* < .05).

Thus, when we grouped oral communication and combined communication in one category, there were significant differences in mode of communication by age at implantation.

In order to evaluate the contribution of the study variables to mode of communication for the entire sample, we conducted logistic multi-nominal regressions. All the study variables were entered into the regressions. Districts were grouped into two categories: the periphery consisting of the northern and southern districts and the central area consisting of all the other districts. Table 3 presents the factors contributing to mode of communication among the entire sample, while the dependent variable appears as two dummy variables: combined communication and manual communication verses oral communication (as a comparison group). The independent variables were: gender (female vs. male), age (25–30 vs. 18–24), ethnicity (ultra-Orthodox and Arab vs. the general population), district (periphery verses center), assistive device (CI and hearing aids vs. no device), and first degree relatives with HL (presence of first-degree relatives with HL vs. no relatives with HL).

**Table 3:** Main factors contributing to mode of communication among the entire sample (*N* = 1210): Results of logistic multi-nominal regressions

Dependent variable:	Combined communication vs. oral communication			Manual communication vs. oral communication		
Independent variables:	B	Odds ratio	CI 95%	B	Odds ratio	CI 95%
Gender (vs. male):						
Female	0.01-	0.99	0.74–1.34	*0.41-*	0.67	0.47–0.93
Age (vs. 18–24):						
25–30	**0.42-	0.66	0.48–0.89	0.03	1.04	0.73–1.47
Ethnicity (vs. general):						
Ultra-Orthodox	**1.80-*	0.17	0.08–0.33	**2.62-*	0.07	0.02–0.32
Arab	**0.72*	2.04	1.40–2.99	**1.42*	4.13	2.79–6.13
District (vs. center):						
Periphery	0.07	1.08	0.74–1.56	**0.70*	2.01	1.36–2.96
Assistive device (vs. no device):						
CI	**1.33-*	0.26	0.13–0.54	**3.41-*	0.03	0.02–0.07
Hearing aid	**1.01-	0.36	0.18–0.72	**2.73-*	0.07	0.04–0.12
First degree relatives with HL (vs. No):						
Yes	**0.49	1.63	1.18–2.24	**0.74*	2.08	1.44–3.02
Pseudo R Square:						
Cox and Snell	0.370					
Nagelkerke	0.417					
McFadden	0.211					

*p* < .001*** *p* < .01** *p* < .05*

As shown in Table 3, female gender made a significant negative contribution to use of sign language. Older age made a significant negative contribution to combined communication. Ultra-Orthodox ethnicity and use of a CI or hearing aid made a significant negative contribution to use of sign language and combined communication. Arab ethnicity and having a first-degree relative with HL made a positive contribution to use of sign language and combined communication. Living in the peripheral districts made a positive contribution to use of sign language.

In order to evaluate the contribution of study variables to mode of communication among the sub-group of participants using a CI, we conducted logistic multi-nominal regressions. Table 4 presents factors contributing to mode of communication among participants using a CI. As in Table 3, the dependent variable appears as two dummy variables: combined communication and manual communication verses oral communication (as a comparison group). The independent variables were similar to those in Table 3, with the addition of age at implantation (age 3+ vs. under age 3) and the omission of an assistive device.

**Table 4:** Main factors contributing to mode of communication among participants using a CI (*N* = 386): Results of logistic multi-nominal regression

Dependent variable:	Combined communication vs. oral communication			Manual communication vs. oral communication		
Independent variables:	B	Odds ratio	CI 95%	B	Odds ratio	CI 95%
Gender (vs. males):						
Females	0.29-	0.75	0.46–1.22	**0.87-	0.42	0.21–0.85
Age (vs. 18–24):						
25–30	*0.62-	0.54	0.32–0.91	0.05-	0.95	0.47–1.94
**Ethnicity** (vs. general):						
Ultra-Orthodox	**1.53-*	0.22	0.10–0.48	*2.39-	0.09	0.01–0.71
Arab	*0.79	2.21	1.11–4.39	***1.72	5.59	2.47–12.66
District (vs. center):						
Periphery	*0.58	1.78	0.94–3.38	**0.98	2.67	1.22–5.85
Age at implantation (vs. > 3):						
≤3	0.51-	0.6	0.24–1.48	*1.41-	0.24	0.05–1.24
First degree relatives with HL (vs. No):						
Yes	**0.80	2.22	1.32–3.70	*0.64	1.87	0.93–3.83
Pseudo R Square:						
Cox and Snell	0.246					
Nagelkerke	0.287					
McFadden	0.145					

*p* < .001*** *p* < .01** *p* < .05*

As shown in Table 4, among participants with a CI, female gender and a young age at implantation made a significant negative contribution to use of sign language. Older age made a significant negative contribution to combined communication. Ultra-Orthodox ethnicity made a significant negative contribution to the use of sign language and combined communication. Arab ethnicity, living in peripheral districts, and having a first-degree relative with HL made a positive contribution to the use of sign language and combined communication.

## Discussion

The purpose of the present study was to examine the associations between demographic and family variables and mode of communication among young people with HL. Specifically we explored whether mode of communication was associated with ethnicity, place of residence, and hearing status of family members. Additional variables previously reported as related with outcomes of habilitation of children with HL were included as well.

Chi-square tests revealed significant associations between mode of communication and all of the study variables. In addition, all the study variables made a significant contribution to mode of communication. Female gender has been documented in the literature as being associated with better language development among children using a CI [7], as has age at implantation [8, 38–41]. Thus, the present study findings demonstrating significant associations between mode of communication and gender and age at implantation are consistent with previous reports.

Use of an assistive device was also considered to be associated with mode of communication. It is not surprising that the majority of participants in the present study who did not use an assistive device used sign language. On the other hand, it is interesting to note that only nearly 54% of participants with a CI used oral language only. The remainder used either sign language only (14%) or combined communication (32%). Research conducted in Australia reported lower rates of sign language usage among CI users, with 9.3% of children using CI at school and only 8.6% of children using combined communication. Parents reported using some sort of sign language with up to 18% of children who participated in the study [35]. It is possible that the higher rates of sign language usage or combined communication in Israel can be attributed to diversity in communication usage related to ethnicity and geographical residence, as will be discussed later.

To the best of our knowledge, the present study is the first to address the association between ethnicity and mode of communication among young people with HL in Israel. The results revealed that most of the ultra-Orthodox participants used oral language, while the majority of Arab participants used sign language. It is important to note that approximately 18% of Arab participants had a CI while nearly 60% of the ultra-Orthodox participants had one. Thus, the rates of implantation among these two ethnic groups are strikingly different. Nevertheless, ethnicity had a significant contribution to mode of communication among CI users, meaning that despite differences in the CI rates among different ethnicities, the ethnic background of individuals plays an important role in the development of their communication. In discussing the relevance of ethnicity to mode of communication, we will relate first to the ultra-Orthodox participants, and then to the Arab participants.

It has been claimed that illness can provoke stigma in Hasidic communities. The effect and fear of stigma in these communities may impact patient outcome [42]. It is possible that use of sign language also creates stigma, thus leaving use of oral language the only possibility for children raised in this society. In addition, parents belonging to ultra-Orthodox Hassidic sects may not behave independently in matters of health and habilitation, since religious authorities are involved in the decision-making process [42]. It has been reported that at times of health crises, the religious authority, the *rebbe*, enters into the relationship between doctor and patient. The *rebbe* may help patients deal with doctors, or assist in accessing an expensive or prestigious doctor for them [26]. Thus, ultra-Orthodox parents may not make their own decisions as to the most suitable mode of communication for their deaf child; rather they turn to their religious authority and act according to his instructions. In addition, the custom of referring to expensive or prestigious doctors may lead them to specialist professionals, in their efforts to find the best oral communication habilitation, resulting in adequate oral communication abilities.

The striking finding of low use of oral language among Arab participants prompts the question as to inequalities in service delivery across populations. One issue concerns availability of speech-language pathologists and audiologists who are native Arabic speakers. Research emphasizes the importance of delivering medical services for patients in their native language [43]. We assume it is possible that deaf Arab children do not have access to services in their native language, which prevents them from acquiring adequate speech and language proficiency. Hence, the only alternative left for them is sign language, which is commonly used among some Arab communities in Israel [24].

Another issue is use of services by parents of Arab children with HL. As mentioned above, it has been documented that ethnic differences seem to contribute to the inequities in service use [9]. For example, in a study conducted in Philadelphia, Pennsylvania, White children were more likely than children from diverse ethnic backgrounds to receive special education services. Furthermore, Black children were less likely than other children to receive special education services [44]. Another study revealed that ethnic groups in Greece experienced higher degrees of inequity in primary health care [9]. Potential racial disparities in access to early intervention services may continue beyond early intervention and exert a long-term impact on the quality of services received by children from racial or ethnic minority groups as they grow older [13]. Israeli Arabs comprise 30% of individuals with hearing loss in the educational system [45]. Although there is an exceptionally high incidence of HL among Israeli Arab children, research indicates that their parents make far less use of all types of services [46], despite a national health insurance system with universal coverage [47]. Acquisition of speech, language and hearing proficiencies requires intensive habilitation by professionals, and earlier age at intervention is associated with better oral language outcomes [3]. A recent study conducted in Israel demonstrated that mothers from the Bedouin population, which is part of the general Arab population, are left to cope on their own with rearing children with HL, without help from their husbands or their community [48]. This situation questions the ability of these mothers to seek habilitation for their children, an issue that deserves further research.

Another unique finding of the present study is the association between place of residence and mode of communication. Significantly lower rates of oral language use were found in the northern and southern districts of Israel. Access to medical professionals was found to be a predictor of families failing to follow up subsequent to diagnosis of HL [12]. It is possible that there is a lack of professional services for children with HL in peripheral areas, meaning that many children residing in these areas are unable to acquire adequate oral language proficiency. Further research is needed in order to examine the availability of services for children with HL in remote areas in Israel. Furthermore, belonging to a minority group and living in a rural area may have particular implications for access to health care [49]. Thus, Israeli Arabs living in remote areas might be vulnerable to greater inequalities in health care, resulting in greater disparities in outcomes of children with HL with this background. This could be the consequence of both lack of services and limited education [50]. Indeed, research indicates that racial/ethnic disparities in child health are linked to an unequal geography of opportunity rooted in residential segregation [51]. In fact, geographic variations in health care have been found to be responsible for a substantial component of observed racial disparity in care [52]. Further research is needed in order to explore differences in communication modes among Israeli Arabs with HL in relation with place of residence.

Finally, the literature relates to the association between the hearing status of parents and mode of communication of their deaf or hard-of-hearing children [34]. In the present study, we related to hearing status of first-degree relatives of participants, including siblings. More than 50% of the participants in the present study had first-degree relatives with HL. The majority of children with HL are born to hearing parents, whereas only 5–10% of them are born to deaf or hard-of-hearing parents [53]. Thus, it is logical to conclude that many of the first-degree relatives with HL in the present study were siblings as well as parents. Research usually does not relate to siblings as a possible variable contributing to mode of communication used by young people with HL. The present finding of a significant association between the hearing status of first-degree relatives and mode of communication is unique to this study. This association deserves further exploration, as in the present study we did not differentiate between the hearing status of siblings and of parents. Future studies should separate the hearing status of parents from that of siblings to deepen understanding of the relationship between the hearing status of siblings and mode of communication of young people with HL.

The findings of the present study underline the need for better monitoring of Arab children with HL and children residing in peripheral areas in Israel and for improving access to habilitation services. Three aspects of habilitation have to be considered. First, services should be culturally and linguistically sensitive. Israeli Arab children should receive speech and language habilitation by Arabic-speaking professionals. Secondly, health service providers have to ensure adequate geographical distribution of services for children with HL. Finally, health education for diverse populations has to be a major goal of policymakers. Further research focusing on the barriers to utilizing habilitation services may provide additional policy directions.

The present study has several limitations. First, the data in this study was abstracted from files and database with the possibility of reporting bias. In addition, since the study was conducted in Israel, its results cannot be generalized to the entire population of young people with HL. Nevertheless, it sheds light on the role that ethnicity and place of residence play in habilitation of children with HL. Another limitation of the present study in not including educational settings as a variable related to mode of communication. Educational setting is a main variable in the foundation of mode of communication among children with HL. This variable was not included in the present study, since this information was lacking. It is possible that Arab children mainly have the opportunity to join only sign-language educational settings, influencing their mode of communication choices. In future studies it is recommended to include educational settings of participants as one of the variables contributing to mode of communication among people with HL. Finally, habilitation of participants in the present study occurred many years before the conduction of the study. It is possible that habilitation services have changed since then, resulting in changes in distributions of modes of communication among young children with HL in Israel.

## Conclusion

Ethnicity and geographic place of residence in Israel are associated with habilitation outcomes of young people with pre-lingual HL. The findings of the present study underline the need for better monitoring of Israeli-Arab children with HL and children residing in peripheral areas in Israel and for improving access to habilitation services. The findings of the present study make an important contribution to understanding disparities in outcomes of pre-lingual HL.

## Data Availability

The datasets used and analyzed during the current study are available from the corresponding author on reasonable request.

## Abbreviations

CI: Cochlear implant
HL: Hearing loss
MOLSASS: Ministry of Labor, Social Affairs and Social Services
